# Balancing Academics and Life: Qualitative Study of Health Professions Students’ Perceptions of a Four-Day Academic Week in the United Arab Emirates

**DOI:** 10.2196/67775

**Published:** 2025-11-04

**Authors:** Ashokan Arumugam, Jacqueline Maria Dias, Sangeetha Narasimhan, Raneen Mohammed Qadah, Reime Jamal Shalash, Taif A M Omran, Bashair M Mussa, Basema Saddik, Nadia Rashed Al Mazrouei, Sivapriya Ramakrishnan

**Affiliations:** 1 Department of Physiotherapy, College of Health Sciences, University of Sharjah, PO Box 27272, Sharjah, United Arab Emirates, 971 6 505 7323; 2 Neuromusculoskeletal Rehabilitation Research Group, RIMHS – Research Institute of Medical and Health Sciences, University of Sharjah, Sharjah, United Arab Emirates; 3 Sustainable Engineering Asset Management Research Group, RISE - Research Institute of Sciences and Engineering, University of Sharjah, Sharjah, United Arab Emirates; 4 Department of Physiotherapy, Manipal College of Health Professions, Manipal Academy of Higher Education, Manipal, Karnataka, India; 5 Department of Nursing, College of Health Sciences, University of Sharjah, Sharjah, United Arab Emirates; 6 Department of Oral and Craniofacial Health Sciences, College of Dental Medicine, University of Sharjah, Sharjah, United Arab Emirates; 7 Department of Basic Medical Sciences, College of Medicine, University of Sharjah, Sharjah, United Arab Emirates; 8 Department of Public Health and Epidemiology, College of Medicine and Health Sciences, Khalifa University, Abu Dhabi, United Arab Emirates; 9 School of Population Health, Faculty of Medicine and Health, University of New South Wales, Sydney, Australia; 10 Department of Pharmacy Practice and Pharmacotherapeutics, College of Pharmacy, University of Sharjah, Sharjah, United Arab Emirates

**Keywords:** four-day academic week, students, academic performance, work-life balance, support systems, United Arab Emirates

## Abstract

**Background:**

Since January 2022, Sharjah in the United Arab Emirates has implemented the 4-day work week model for the first time in the public and private sectors, including universities. While this framework may enhance productivity and work-life balance for many professionals, the current study specifically explores the perceptions of students in medicine, dentistry, and health sciences programs regarding the impact of transitioning from a 5-day to a 4-day work week on their academic performance.

**Objective:**

The objective of this study was to explore and analyze the perceptions of students in medicine, dentistry, and health sciences regarding the implementation of a 4-day academic week in the Emirate of Sharjah, United Arab Emirates.

**Methods:**

Twenty-four university students (mean age 20.95, SD 1.30 years; 12 men) studying in medicine, dentistry, physiotherapy, nursing, or medical diagnostic imaging programs, who experienced a transition from a 5-day week to a 4-day week, participated in semistructured interviews lasting approximately 20‐30 minutes. All interviews were recorded and transcribed verbatim. The Braun and Clarke 6-phase framework of thematic analysis was used.

**Results:**

We identified 5 themes: academic journey, academic work-life balance, support systems, classroom dynamics, and common stressors of the 4-day academic week. Overall, most students reported increased motivation, engagement, and academic achievement following the transition from a 5-day to a 4-day week. In addition, participants described a positive academic work-life balance, improved physical and mental well-being, optimal use of time for both academic and personal commitments, favorable support from faculty and family members, and maintained or even improved attendance levels. Nevertheless, some students expressed concerns about condensed schedules and longer days, increased stress, disrupted work-life balance, and inadequate support systems to cope with this new framework.

**Conclusions:**

Overall, the 4-day academic week enhanced motivation, academic performance, work-life balance, and the physical and mental well-being of medicine, dental, and health science students. However, some students experienced challenges related to condensed schedules and increased stress. These mixed outcomes highlight that while the 4-day work week offers notable advantages, careful planning and support are essential to mitigate the potential drawbacks and ensure all students can succeed within this new academic framework. Future research could explore strategies to address these challenges and further improve the 4-day week experience for all students.

## Introduction

The concept of a 4-day work week has gained significant attention, particularly in the aftermath of the global pandemic [1]. This shift has sparked a movement toward reducing the traditional 5-day work week to 4 days across various industries, including higher education. Typically, universities operate on a 5-day schedule, but with the adoption of a 4-day week, classes are now condensed into 4 days, which allows a long (3-day) weekend. Numerous studies have highlighted the advantages of this model, such as enhanced productivity, improved job satisfaction, better academic work-life balance, lower stress levels, and reduced personal expenses, such as commuting costs [2,3].

The 4-day school week was introduced in a South Dakota school in 1936. In 1973, many schools across the northeastern United States adopted this model to address economic challenges, particularly those related to energy and operational costs [4,5]. In more recent years, the number of school districts transitioning from the traditional 5-day week to a 4-day week with extended hours has increased [6]. Hewitt and Denny [4] reported minimal differences in the academic performance of students attending schools on the 5-day schedule compared to those following the 4-day system. Furthermore, this study recommended considering multiple factors beyond academic performance when evaluating the effects of the 4-day work week model [4].

According to Sagness et al [7], the 4-day school week offers potential advantages for both rural and suburban schools, particularly in terms of student achievement, behavior, attendance, as well as job satisfaction among teachers and staff. Thompson and Ward [6] revealed that students in nonrural 4-day schools had a reduction in on-time grade progression and absenteeism with the implementation of 4-day week. However, a few United States district schools reverted to a 5-day week due to reduced academic achievement [8].

A recent study found that school schedules during a 4-day school week have had detrimental impacts on student achievement, with a decline in math and reading scores [9]. On the contrary, another study conducted on students found that there is a positive correlation between implementing a 4-day school week and academic performance in reading and mathematics [10].

Since January 2022, Sharjah, one of the 7 Emirates in the United Arab Emirates, has adopted the 4-day (work) week model across both the public and private sectors. This happened after the United Arab Emirates moved its weekend from Friday-Saturday to Saturday-Sunday, mainly for governmental institutions, to better align its business economy with global markets. A government study, presented to the Sharjah Executive Council in 2023, revealed significant improvements in job performance, happiness, and mental well-being among the government employees [11]. A survey conducted in 2023 among 40,000 parents and employees of schools by the Sharjah Private Education Authority revealed that school children’s absenteeism decreased, motivation increased, and behavioral problems reduced while teachers’ work-life balance improved significantly [12]. A recent survey analyzing the impact of a 4-day academic week on dental students in the United Arab Emirates revealed high levels of satisfaction with both their academic and clinical training. Students expressed contentment with the extended weekend, which they found beneficial for reducing stress, improving exam preparation, and allowing them to spend more time with family and friends [13]. However, only dental students with or without the experience of both 4-day week and 5-day week were included in this survey. These findings cannot be generalized to all medicine and health sciences students, as their coursework, clinical practice, and laboratory skills requirements are relatively different. In addition, those students who have not experienced a transition from a 5-day week to a 4-day week may not be able to appreciate the benefits and challenges of this changeover. Although electronic surveys are easy to administer, cost-effective, and quick to deploy, they present challenges such as difficulty in identifying the characteristics of nonrespondents and maintaining confidentiality, among others. They do not provide the same in-depth understanding of participants’ perceptions, actions, thoughts, and experiences as (semistructured) interviews do [14].

Although numerous studies have investigated the effects of a 4-day academic or work week in schools and various sectors, no research has specifically examined its impact on university students in medicine, dental, and health sciences programs who transitioned from a 5-day to a 4-day academic week. Therefore, it is crucial to assess how this shift affects the academic performance of students in these programs. To the best of our knowledge, this is the first qualitative study of the perceptions of medicine and health sciences students in Sharjah regarding the 4-day work week model using semistructured interviews. If the 4-day work week is found to improve academic outcomes, the results could have broader implications for educational institutions still operating on a 5-day work week, prompting them to consider adopting a 4-day work week schedule to support student performance in the other 6 Emirates. The themes and insights emerging from this study will provide valuable perspectives on how the 4-day work week impacts the academic performance of students in medicine, dental, and health sciences programs.

## Methods

### Study Design, Setting, and Participants

In this study, we adopted an exploratory qualitative research design using semistructured interviews. A qualitative approach enabled the understanding of the perceptions of students who had used both the 5-day week and were not exposed to the 4-day week. We adhered to the COREQ (Consolidated Criteria for Reporting Qualitative Studies) guidelines for reporting this qualitative research [15].

### Study Population

All students enrolled in the medical and health sciences colleges at the University of Sharjah, United Arab Emirates, were invited to participate in the study through advertisements posted on university notice boards, emails, and word of mouth. The participants included students who had experienced the transition from a 5-day week to a 4-day week, with the study taking place during the Fall semester of 2023. These students had been exposed to 3 semesters since the introduction of the 4-day work week. Students were selected based on their willingness and availability to participate. Purposive sampling was used to ensure representation from the 4 different colleges on the medical campus, including medicine, dental medicine, pharmacy, and health sciences. Recruitment of participants continued until data saturation was reached. The interviews were conducted in one of the classrooms on the medical campus, free from noise and distractions.

### Instrument

An electronic search of PubMed, Scopus, and Google Scholar was conducted to inform the development of interview questions. We used the following search terms: (“student” OR “learner” OR “pupil” OR “scholar”) AND (“perception” OR “attitude” OR “view” OR “opinion”) AND (“transition” OR “change” OR “shift” OR “move”) AND (“four-day” OR “4-day” OR “four day”) AND (“five-day” OR “5-day” OR “five day” OR “traditional”) AND (“academic week” OR “school week” OR “educational week” OR “work week”). We could not find qualitative studies on university students’ perceptions of transitioning from a 4-day to a 5-day academic week. Only one survey published in 2024 has reported the impact of a 4-day week on academic performance and study–life balance of university students studying dental medicine [13]. To inform the development of the interview guide, we reviewed existing literature on the transitions in education, school and university students, and alternative workweek models in other sectors [6,13,16-19]. Although some studies were based on survey designs rather than qualitative studies, they provided relevant prompts, clues, and contextual insights that shaped the structure and content of our questions tailored to answer our aims of the study.

Our interview questions and probes were explicitly designed to align with the study’s specific research aims, allowing a focused exploration of participants’ perspectives that are consistent with our objectives. Two independent external content experts with qualitative research experience—one a physiotherapy academic from India and the other a nursing academic from the United Arab Emirates, both not involved in the study—reviewed the interview guide and suggested minor amendments. The interview guide was piloted during mock interviews prior to data collection to ensure clarity. The final interview questions are presented in Textbox 1.

Textbox 1. **Semistructured interview questions.**

Questions

1. In your opinion, would you tell me about the impact of a 4-day workday week on your lectures and lab sessions?
  - How would you describe your current attendance levels in lectures and labs compared to a 5-day week?
  - In your opinion, are there any specific changes in the method of lectures and lab taught?
  - Would you like to describe any specific consequences or concerns over the 4-day work week in your academic life?
2. In what ways has the 4-day work week impacted your assignments and coursework?
  - In what ways has the 4-day week impacted individual and group assignments compared to a 5-day week?
  - In your opinion, were there any specific changes in the type of assignments and course work compared to a 5-day week?
3. In what ways has the 4-day work week impacted on learning theoretical information and honing practical (lab or clinical) skills?
4. In what ways did the 4-day work week affect your performance in mid-term and final exams?
  - How do you feel that the 4-day work week has affected your ability to prepare for exams or complete assignments in a timely manner?
5. How did the 4-day work week affect your clinical placements and gaining clinical skills?
6. Would you share with us some strategies which you adopted to maintain your academic performance during a 4-day week compared to a 5-day week?
7. Could you describe the support or help you would have received from your faculty, department, and college to support your academic performance following the transition to a 4-day weekday week?
  - What external help or support have you received?
  - How effective was the help or support? In what ways did you benefit from the support?
8. Can you tell me how you feel about the 4-day work week in terms of your academic performance? Has it improved or gone down?
9. In your opinion, what are the benefits and drawbacks of having a 4-day work week on your academic performance?
10. How would you describe any changes in your motivation or level of engagement with your coursework or lab sessions since the implementation of the 4-day work week?
11. How do you think the 4-day work week has affected your ability to balance your academic workload with other commitments and responsibilities?
12. Do you think there’s anything else I can do to understand your academic performance during a 4-day week better, but I have not asked?

### Collection of Data

This qualitative research relied on collecting data directly from students using an exploratory approach, which enabled an in-depth understanding of their perception of the 4-day work week and its impact on their academic performance [20]. Semistructured interviews are a well-established method in qualitative research. Each interview lasted between 20 and 30 minutes. The interviews were recorded after obtaining written informed consent from all participants. Two female team members (RMQ and RJS) who are graduate research assistants with master’s degrees of science in physiotherapy received training in qualitative interview techniques from an expert researcher in the research team (JMD) who was experienced in conducting qualitative interviews. The first 4 interviews were conducted in the presence of JMD, after which RMQ and RJS conducted the remaining interviews independently. They wrote field notes to facilitate data analysis. They recorded all interviews and transcribed them verbatim in English. Throughout the interviews, participants were encouraged to ask questions or express doubts to ensure clarity and reduce ambiguity. The research team members were always available to answer any questions from students and provide clarifications where required. All transcripts were sent back to the students to ensure accuracy of the data captured and to ensure member checking. A range of viewpoints was captured, and data saturation was achieved.

### Data Analysis

Thematic analysis was used to analyze the data. The interviews were transcribed verbatim by two members of our team (RMQ and RJS). The 6 phases of thematic analysis as defined by Braun and Clarke were followed [21] : (1) familiarizing oneself with the data, (2) generating codes, (3) constructing themes, (4) reviewing potential themes, (5) defining and naming themes, and (6) producing the report. Two investigators (AA and JMD) initially read the transcripts to generate codes and to document where and how patterns occurred. They performed data reduction, and they collapsed data into labels to create categories to facilitate efficient analysis. Six team members (AA, JMS, RMQ, RJS, SN, and SR) were involved in an iterative and analytical process to develop themes from the initial codes. We identified patterns (differences and similarities) in the data by repeatedly reading the transcripts and memos. The use of memos throughout the data analysis promoted reflexivity, precluded a priori biases, and ensured the credibility of our analysis and associated findings. Themes were revised through constant comparison across transcripts, and an audit log was maintained to document data-driven decisions and ensure transparency. All team members reviewed the data to ensure consistency and coherence between the raw data, the generated codes, and the resulting themes. Representative quotations were used to support the themes, ensuring transparency, and justification of interpretations in the data.

### Team Reflexivity

Our research team included members from diverse disciplinary backgrounds, including physiotherapy, nursing, medicine, dental medicine, pharmacy, public health, medical education, and qualitative research. Moreover, the team’s experience levels ranged from research assistants and lecturers to senior academics and professors. Our multidisciplinary approach with variations in roles and expertise allowed for a rich interpretation of the data while promoting reflexivity throughout the study. All investigators acknowledged and discussed their own professional perspectives, assumptions, and potential biases prior to data collection and during the thematic analysis process.

To enhance reflexivity, memos were used throughout data analysis to document emerging thoughts, evolving decisions, and reflections. Regular team meetings were held to critically examine how our positionalities and prior experiences might influence coding and theme development. Two research assistants not involved in teaching responsibilities conducted the interviews to mitigate the influence of faculty on students and social desirability bias. We involved two external content experts in reviewing the interview guide, further improving the objectivity of our semistructured interview guide.

### Ethical Considerations

The study was performed in line with the principles of the Declaration of Helsinki. Ethical approval was granted by the University of Sharjah Ethics Committee (reference number: REC‐23‐06‐13‐01‐F). Informed consent was obtained from all the participants who were assured of the anonymity of their responses and that their participation in this research would not affect their course grades. Each participant was assigned a random, alphanumeric code to ensure confidentiality. Interview content was analyzed by the research team, and data were securely stored with the primary investigator (AA) on a password-protected computer. The data will be retained for 5 years, after which it will be destroyed.

## Results

### Overview

In total, there were 24 participants with equal male and female representation in the health professions. All 4 colleges at the medical campus of our university were represented, including medicine, dentistry, pharmacy, and health sciences. The demographics of the study participants are listed in Table 1.

**Figure 1.**
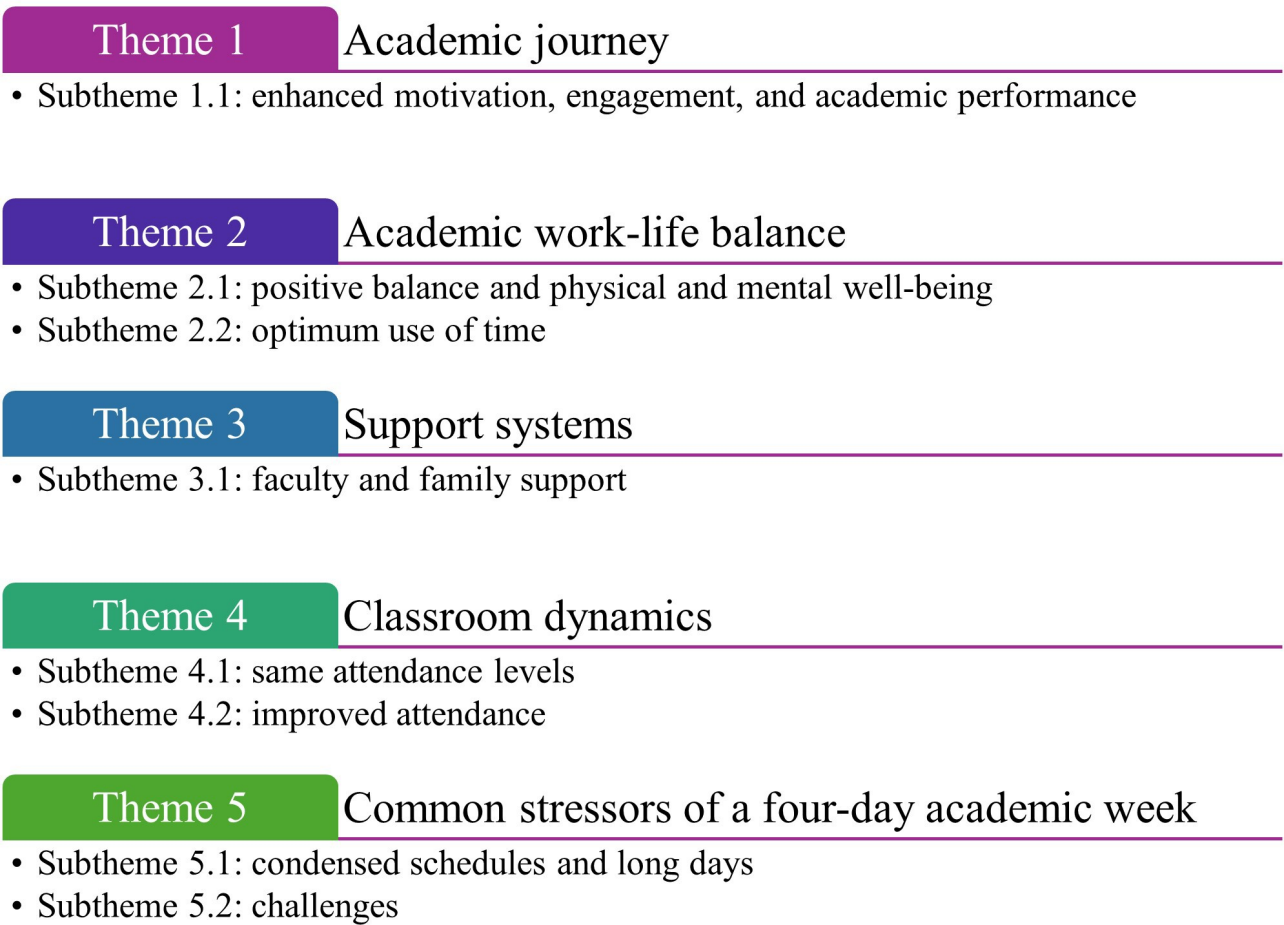
A summary of themes and subthemes reflecting medicine, dentistry, and health sciences students’ perspectives on the impact of the transition from a 5-day to a 4-day week on their academic performance.

The findings from this study offer a wealth of valuable data. Students’ perspectives were grouped into 5 main themes. Each theme was further divided into one or more subthemes. Figure 1 and Multimedia Appendix 1 summarize the themes and subthemes derived from the students’ responses.

**Table 1.** Demographic characteristics of participants.

Participant ID	Age (years)	Sex	College	Program of study
P1	20	F^a^	Health sciences	Medical Diagnostic Imaging
P2	21	F	Health sciences	Nursing
P3	22	M^b^	Health sciences	Nursing
P4	20	M	Health sciences	Physiotherapy
P5	20	M	Health sciences	Physiotherapy
P6	21	F	Health sciences	Physiotherapy
P7	20	M	Medicine	Medicine
P8	20	F	Medicine	Medicine
P9	19	F	Medicine	Medicine
P10	19	F	Medicine	Medicine
P11	20	M	Medicine	Medicine
P12	19	M	Medicine	Medicine
P13	23	M	Dentistry	Dentistry
P14	22	M	Dentistry	Dentistry
P15	23	M	Dentistry	Dentistry
P16	23	M	Dentistry	Dentistry
P17	22	M	Dentistry	Dentistry
P18	22	M	Dentistry	Dentistry
P19	22	F	Dentistry	Dentistry
P20	22	F	Dentistry	Dentistry
P21	22	F	Dentistry	Dentistry
P22	20	F	Pharmacy	Pharmacy
P23	20	F	Pharmacy	Pharmacy
P24	21	F	Pharmacy	Pharmacy

a F: female.

b M: male.

### Theme 1: Academic Journey

#### Subtheme 1.1: Enhanced Motivation, Engagement, and Academic Performance

The implementation of a 4-day academic week has notably enhanced students’ motivation, engagement, and academic performance, as expressed by many health sciences students. Many students reported a marked increase in motivation, attributing it to having more time to prepare for exams, complete assignments, and organize their studies.

*Since the implementation of the four-day week, my motivation levels increased. Having an extra weekend day will help you have more time to prepare for upcoming exams and complete assignments on time*.

[P6]

One student added that additional time during the weekend improved his grades.

*My grades got better because I had more time to study.*

[P13]

Some students observed that with more time to rest during the extended weekend, they returned to their studies feeling more focused and refreshed. The extra day off also encouraged greater self-reliance for one’s own learning, with students feeling more motivated to study independently rather than relying solely on instructors. Furthermore, the 4-day work week not only helped them stay engaged but also made them more productive, providing a sense of balance and convenience that positively impacted their academic efforts.

### Theme 2: Academic Work-Life Balance

#### Subtheme 2.1: Positive Balance and Physical and Mental Well-Being

A significant number of study participants reported that the transition to a 4-day academic week markedly improved their academic work-life balance. Many appreciated that the additional day off allowed them to allocate more time to their studies, extracurricular activities, and personal commitments. Students highlighted that the extra time enabled them to better manage academic workload and responsibilities, while also dedicating time for rest, exercise, family gatherings, and social interactions.

*As I mentioned, because now I have one extra day, I can enjoy during the weekend, I can distribute the work better, so I have better actual balance between both my studies and extracurricular (activities).*

[P5]

The resident students in the dormitory found the extended weekend particularly beneficial for traveling back to their families.

*So, it’s easier to travel to your family outside the country.*

[P21]

Some students noted that having an additional day to themselves contributed to better mental health, allowing for reflection, relaxation, and a reprieve from the daily academic pressures. While the 4-day schedule condensed the lab and lecture sessions, students acknowledged that the 3 days off helped them recharge and maintain a healthy balance between their academic and personal lives. Overall, the shift to the 4-day academic week was seen as highly beneficial in promoting both academic productivity and overall well-being.

#### Subtheme 2.2: Optimum Usage of Time

The shift to a 4-day academic week had a positive impact on the students’ ability to manage and optimize their time. Many expressed that under the previous 5-day schedule, there was insufficient time to complete coursework, particularly clinical documentation, which often had to be completed at home. With the additional day off, students found they had more time to focus on assignments, manage their academic workload, and balance extracurricular activities. An extra weekend day allowed for better organization of tasks, leading to improved time management and more efficient studying.

*Having the three-day weekend helped me organize my time better.*

[P7]

*Having an extra weekend day allowed me to balance both workload and commitments, helping me manage my time better.*

[P11]

In addition, students noted that the 3-day weekend gave them more energy and focus during the week, as they had more time to rest and recharge. Some also emphasized that the extra day helped them achieve a better balance between academic responsibilities and personal commitments, including spending time with family, which had been challenging with the previous schedule. In summary, a considerable proportion of the university students noted a major improvement in terms of time management and productivity after shifting to the 4-day academic week.

### Theme 3: Support Systems

#### Subtheme 3.1: Faculty and Family Support

Health care students expressed a strong sense of support from both faculty and family, which played a pivotal role in their academic success and well-being. Many students highlighted the encouragement they received from specific faculty members who were not only understanding but also flexible in accommodating their needs. Whether it was adjusting lab timings, rescheduling exams, or providing additional resources such as opening labs on weekends, faculty demonstrated a commitment to helping students balance their academic and personal responsibilities. One student mentioned how faculty members worked around their professional commitments, enabling them to complete a movie shoot while staying on track academically.

*Yes, from Doctor xxxx (a faculty member’s name) and Doctor xxxx (another faculty member’s name) as well; basically, in one of the semesters, I had Netflix shoot, and I was going to be absent from university for two months in a row. I talked with Doctor xxxx (a faculty member’s name) as she’s my advisor and she helped me out, also Doctor xxxx (another faculty member’s name) helped me and they made it possible for me to do my shooting, to do my work and to attend my makeup (exams) better.*

[P3]

Moreover, the emotional and motivational support from family and friends further empowered students to stay focused and motivated, allowing them to better organize their priorities and excel in their studies. Based on the perceptions of the 4-day academic week, it can be inferred that the combined support from faculty, family, and peers significantly enhanced the students’ academic experience, making the 4-day academic week more manageable and effective whilst attending to personal responsibility outside of academia.

### Theme 4: Classroom Dynamics

#### Subtheme 4.1: Same Attendance Levels

Nearly 70% of the participants reported that their attendance levels did not alter in the shift from 5-day week to 4-day academic week.

#### Subtheme 4.2: Improved Attendance

Around 25% of the study respondents recorded that their attendance got better, and they have started attending all the classes with the 4-day academic week, while before they used to stay absent or attend the maximum number of required classes and clinical practicums.

### Theme 5: Common Stressors of a 4-Day Academic Week

#### Subtheme 5.1: Condensed Schedules and Long Days

The extended working hours and condensed schedules brought about by the shift to a 4-day academic week were one of the major concerns raised by the health care students. Many found that the teaching content was compressed into fewer days, leaving minimal time for breaks or adequate reflection.

*The negatives of having four days are that the materials are compressed into these four days, leaving minimal time.*

[P1]

*But on the other side, the drawbacks for having 4 days is (are) that we usually have a lot of materials that’s compressed on us in these four days that we have minimal time.*

[P5]

Some students noted that their weekdays became more stressful, with longer hours and more back-to-back lectures, making it difficult to manage their workload effectively. The compact schedule, though allowing for a longer weekend, resulted in longer days, leading to increased fatigue and diminished focus during lectures.

*The days became longer and compact. It became more stressful because the days became longer.*

[P4]

Despite these challenges, some students accepted the trade-off, stating that they valued the extra day off on weekends, even though the compressed weekdays required more intense time management.

*The drawback is for example, nowadays the classes are many; let’s say for example today is Monday, so I started at 8:00 (am) and I finished at 4.00 (pm), but before I used to start at 10 and then I would finish that too (at 4.00 pm). We have more time to divide the classes, but for me personally, and I think most people will agree with me, it’s fine to have everything compacted on the weekdays as long as on the weekend we have an extra day off.*

[P3]

Therefore, the main drawback of the 4-day academic week appeared to be the increased workload on each individual day, which students found challenging to manage, despite the perceived benefits of extended weekends for relaxation and study.

#### Subtheme 5.2: Challenges

While the 4-day academic week was beneficial to the health care students, many of them also voiced several challenges associated with this model. Some noted that the longer weekend led to a tendency to neglect studies during the break, resulting in lapses in focus or forgotten material.

*…. but the drawbacks that we may forget things we may like that we will not study in these three days and sometimes it happened.*

[P1]

The compacted schedule caused others to arrive late to lectures or struggle with back-to-back classes, which they found overwhelming.

*I’m arriving late to the lectures by around 15 minutes because of the back-to-back classes.*

[P2]

Several students reported a decline in their grades, attributing it to increased stress and the tutors’ inability to cover all the material during class time, leaving more for self-study.

*My grades got decreased due to stress.*

[P9]

*but for the negative impact, as I mentioned, the tutors are unable to finish materials. So, there are a lot (of) self-study material.*

[P10]

Balancing personal responsibilities, family time, and academic workloads became harder for many, as they found it difficult to manage their time effectively within the condensed 4-day academic week. Though the extended weekend was considered a benefit, it often came at the cost of a worsened balance between academic and personal responsibilities.

## Discussion

### Principal Findings

This study revealed that the shift to a 4-day academic week from a 5-day week had a significant impact on various aspects of our students’ academic and personal lives. We found that the 4-day academic week had a positive impact on most of our student participants, enhancing their motivation, engagement, academic work-life balance, and physical and mental well-being. Some students received positive support from faculty and family. However, a few students encountered increased stress and struggled to balance academic and personal commitments with this model.

Most participants reported increased motivation, focus, and engagement in both lectures and laboratory sessions, which positively impacted their academic performance. These findings align with the study by Gaballah et al [13] where most students acknowledged improvements in academic performance with the 4-day academic week, although one-third did not report such benefits.

The shift to a 4-day academic week improved students’ academic work-life balance, allowing them to allocate more time to rest, personal commitments (with family and friends), and extracurricular activities. The extra day off provided an opportunity for reflection and relaxation, which contributed to better mental health and well-being. These findings agree with Gaballah et al [13], who reported that around 90% (257 out of 284) of their dental student participants reported stress relief and spending quality time with friends or families, in addition to having extra time for studies and exam preparation because of the long (3-day) weekend [13]. Dormitory students particularly appreciated the reduced need to travel frequently (considering an extra day off), which decreased physical exhaustion. Many participants emphasized that they could now distribute their academic workload more evenly, avoiding burnout, which is supported by the theory of self-regulated learning**,** which focuses on students’ ability to manage their own learning processes [22]. The improved balance between academic and personal responsibilities not only boosts productivity but also helps students maintain a healthier lifestyle [23]. However, managing this balance required effective time management and self-care to avoid procrastination during the longer weekends, as some students feel that a 4-day academic week has led to academic overload [24-26]. Academic overload may be regarded as students’ feelings of being overwhelmed by their academic requirements or responsibilities while pursuing a degree at university.

While the students enjoyed a 4-day academic week with the exemption of commuting time on an extra day, the long workday and compact schedule took its toll on some students. Entry-level students in higher education have been reported to experience difficulties in managing the academic workload at university [27]. Chambel et al [28] found that students’ inability to manage academic workload had a negative impact on academic adjustment to university and academic performance. Some students were happy with the support received from faculty and family, which would have played a pivotal role in helping students adapt to the 4-day academic week. Indeed, some faculty members were flexible in accommodating students’ needs, offering extra lab time or adjusting deadlines to help them balance their academic and personal responsibilities. The findings show that faculty members played a key role in providing cognitive flexibility by helping students adjust to the new schedule and commitments while maintaining the professorial standards [29]. However, one-third of the dental students, from the previous survey conducted at our institution, reported limited time to meet their professors and advisors, while the rest expressed satisfaction with the support provided by faculty [13].

Many students appreciated the flexibility offered, such as optional assignments and extended lab hours. These arrangements could be beneficial for students who had other professional obligations to improve their academic achievement [30,31]. Emotional support from family also encouraged students to stay focused and motivated. With this support system in place, students felt more confident in managing the demands of their studies. The combined support from faculty, family, and friends might have alleviated much of the stress associated with the compressed schedule, helping students navigate challenges more effectively.

Despite some students receiving favorable support, a few participants reported a lack of adequate support from faculty to facilitate their transition to this new framework. Universities using a 4-day academic week model need to improve manual (eg, student advisors, counselors) and digital support systems (through a dedicated service desk), amongst others, to help mitigate or alleviate the stressors and enhance the academic performance of students adjusting to this transition from a 5-day week.

For most students, attendance levels remained unchanged after the shift to a 4-day academic week, indicating that the change did not negatively affect class participation. All faculty members use an online attendance tracking system at our institution, which issues automatic warnings when students reach 10%, 15%, and 20% absence thresholds. Those exceeding the 20% absence limit are prohibited from attending exams, ensuring consistent attendance and academic engagement. However, a minority of students reported improved attendance, attributing this to increased motivation and better preparation. The extra day off might allow students to rest and recharge, which would have made them more consistent in attending classes [32]. This improvement in attendance also reflects a stronger commitment to academic schedules and responsibilities [33]. However, for those whose attendance remained the same, the extra day was more of a personal benefit than an academic one.

While the 4-day academic week offered clear benefits, many students found the condensed schedule challenging, especially in the clinical rotations, which is in line with the findings of the earlier study [34]. The longer days, with back-to-back lectures and lab sessions, left little time for breaks or reflection. This intensified workload increased stress and fatigue, making it harder for students to stay focused during classes. The compressed schedule might pose challenges to faculty in covering all the materials included in their course syllabi, and some students pointed out their reliance on self-study in this regard.

A few participants felt a lack of extra support from faculty to adjust to this transition to the 4-day academic week. Despite these difficulties, many students accepted the trade-off, valuing the extended weekend for relaxation and personal commitments. The challenge lay in balancing the intensified academic schedule with the need for rest and fulfilling other (personal) commitments [35]. The mixed findings of our study provide a nuanced understanding of the 4-day academic week’s benefits and drawbacks, particularly in the context of higher education in health care in Sharjah, United Arab Emirates.

Academic overload could lead to lower academic adjustment among university students. This means that those students who feel overwhelmed by their daily academic requirements and responsibilities will have lower academic adjustment. This result is in accordance with previous research by Bitzer et al [27]. Although we did not have access to the grades, this may be a plausible explanation. Students who feel confident about their skills, who are confident about their academic and learning capabilities, and who have a positive attitude or perception toward their abilities will perform better academically and adapt to the academic demands of the university. Moreover, a previous study indicated a positive relationship between self-efficacy and academic adjustment [36].

The more time students allocate toward studying, the higher their level of self-efficacy and the better their academic adjustment. In general, the 3-day extended weekend appeared to be conducive to learning.

### Strengths and Limitations of the Study

A variety of students from the different colleges within the medical campus has resulted in rich data, and they provided interesting perspectives on the impact of the 4-day academic week on academic performance across the different colleges. In addition, the study followed a robust protocol to reduce bias and enhance credibility of the findings and adhered to the COREQ guidelines (Consolidated criteria for Reporting Qualitative research) checklist to improve transparency in reporting.

A limitation of the study is that we had included medicine, dentistry, and health science students only from the University of Sharjah, which may not be generalizable to students studying different programs (eg, engineering, law, arts, and science) and other universities and in the other emirates within the United Arab Emirates. However, at present, the Emirate of Sharjah is the only emirate that has implemented the 4-day academic week for universities.

### Implications and Future Recommendations

The findings clearly illustrate the nature in which the transition to the 4-day week in the United Arab Emirates has occurred, along with the positives and negative side. The themes and trends identified in this work will inform and guide current and future research studies, thereby broadening the scope of our work. Future studies are needed to test whether the identified themes are common in the emirate of Sharjah and beyond.

Given the mixed results, a need for a study skills program to accompany the transition to the 4-day work week is required to mitigate the issues encountered by some students. This would include prioritizing and scheduling academic activities by creating a weekly schedule that allocates dedicated time to both academic and personal activities, along with prioritizing tasks and setting realistic goals for every identified task. Students need to manage time efficiently by breaking tasks into smaller, manageable segments and using tools like time-blocking and to-do lists. These strategies can help students stay organized and in control of both academic and personal lives. Students would need to clearly define boundaries between academic and personal life, which is an area of life skills for the future. Moreover, students can practice self-care for themselves, like meditation, exercise, and hobbies that rejuvenate them mentally and physically. Students should be able to seek help from the faculty and family whenever required to accommodate and balance their expectations and needs in the best way possible. Students need to reflect and adjust as necessary, as their academic demands and priorities may change over time.

This study offers recommendations, including that the 4-day academic week, while working in the favor of most university students, needs to have a supportive mechanism. One such support system is an effective advisory system so that students (advisees) can seek help with study skills and time management from their faculty advisors or mentors to mitigate the drawbacks of the extended hours during the 4-day academic week. Our university already has such an advising system (with each student assigned to a faculty advisor), a disability resource center, and counselors in place to support all students, including but not limited to those at risk of academic probation because of their poor performance in studies, to thrive with the current framework. Moreover, our university has introduced peer tutoring activities and is planning peer advising initiatives to further facilitate this transition from a 5-day to a 4-day academic week in the emirate of Sharjah, United Arab Emirates. Further studies involving students from diverse academic disciplines (eg, engineering, arts, literature, science, and law) and from other universities in the United Arab Emirates that have adopted the 4-day academic week are required to generate wider insights and provide implications for policy.

### Conclusion

Overall, students studying medicine, dentistry, and health sciences programs perceived that the 4-day academic week had a positive impact by enhancing their motivation, engagement, class attendance, and work-life balance, along with the support of faculty and family. However, a few students encountered increased stress and struggled to balance academic and personal commitments with this model. Universities using a 4-day week model need to improve manual (eg, student advisors and counselors) and digital support systems (through a dedicated service desk), amongst others, to help mitigate or alleviate the stressors and enhance the academic performance of students adjusting to this transition from a 5-day week to a 4-day week.

## Supplementary material

10.2196/67775 Multimedia Appendix 1 A summary of themes, subthemes, categories, and quotes supporting medical and health sciences students’ perception on the impact of a 4-day academic week on their academic performance.

## Abbreviations

COREQ: Consolidated Criteria for Reporting Qualitative study
