# Supplementary material for: Balancing Academics and Life: Qualitative Study of Health Professions Students’ Perceptions of a Four-Day Academic Week in the United Arab Emirates
Source: JMIR Med Educ. 2025 Nov 4;11:e67775. doi: 10.2196/67775 (PMC12584997; doi:10.2196/67775)
Supplement: Multimedia Appendix 1 [file mededu-v11-e67775-s001.docx]

**Table S1.** A summary of themes, subthemes, categories, and quotes supporting medical and health sciences students’ perception on the impact of a 4-day academic week on their academic performance.

| **Theme** | **Subtheme** | **Participant ID** | **Quote** |
| --- | --- | --- | --- |
| Academic Journey | Enhanced motivation, engagement, and academic performance | P3^a^ | “My motivation has improved, actually, and the good thing about a four-day week is that you don’t notice that the week has finished.” |
|  |  | P5 | “My motivation is now higher, and I depend more on myself to study instead of going immediately to doctors to teach me.”  “As I said, more testing like maybe more responsible for a lot of things at the same time, so it could lead me to be a great person in the future where I could handle a lot of things, you know, less than minimum time than usual.” |
|  |  | P6 | “Since the implementation of the four-day week, my motivation levels increased. Having an extra weekend day will help you have more time to prepare for upcoming exams and complete assignments on time.” |
|  |  | P7 | "Since the implementation of the four-day week, my motivation levels and engagement in my coursework and lab sessions increased. I’ve become more focused, and my grades have improved.”  “The motivation is better because it gives you time for yourself to implement whatever you want.” |
|  |  | P8 | “Convenance (is) better.” |
|  |  | P9 | “Because I will have much time to rest during the weekend, so my focus is better for now.” |
|  |  | P12 | “Since the implementation (of the four-day week), the effort and motivation and level of engagement increased.” |
|  |  | P13 | “My grades got better because I had more time to study.” |
|  |  | P18 | “Because I will have much time to rest during the weekend, so my focus is better for now.” |
| Academic work-life balance | Positive balance and physical and mental well-being | P6 | “It was highly effective to balance my life and academic work, so it was good. The benefit that you have more time on the weekends to study and even to have a balance between, for example, exercising and studying and stuff…..” |
|  |  | P7 | “It helped a lot, because, of course, three days weekend give(s) you more time for other responsibilities or other commitments.” |
|  |  | P10 | “As I mentioned, because now I have one extra day, I can enjoy during the weekend, I can distribute the work better, so I have better actual balance between both my studies and extracurricular (activities).”  “The benefits as a dormitory student, is that I travel every week to my city and I get to have the chance to stay in my city for one extra day, which is actually less physically exhausting….” |
|  |  | P12 | “It’s again better actually having more balance in my life now.” |
|  |  | P13 | “Taking longer vacation (a long weekend break) from studies and gives more time to work on other things.” P13 |
|  |  | P15 | “The benefit is having three weekend days, allowing more time to do assignments, projects, and have time with family and friends.” |
|  |  | P16 | “Now I’m able to balance both my academic workload and personal commitments better.” |
|  |  | P20 | “Having three weekend days is better because I can balance between other commitments like family gatherings and hanging out with friends.”  “I think it allows you to have more time for yourself, like at the end of the week you can actually look back at what happened. You can take (a) deep breath and you don’t need to worry about things like that. Plus, you don’t get to like, you’re not bombarded by people every single day because I feel like in four days, like you can actually have one day to yourself, which is really important.”  “The benefits of this transition (from five-day week to four-day week) gave me more self-time and better mental health.” |
|  |  | P21 | “The benefit is having more time to study and even balance between exercising and studying.”  “So, it’s easier to travel to your family outside the country.”  “The benefits are that you have more time to study, and you have less contact with people on social and environment. Maybe that would also help you feel better and allow socialization with others.” |
|  |  | P22 | “One extra day, it helped in balancing academic workload and family gathering, so it gave more family time.” |
|  |  | P24 | “We had to adjust all the labs and lecture sessions into four days, which was condensed, but at the same time we got three days off, which was a bit relaxing.” |
|  | Optimum usage of time | P3 | “Because before we used to have five days and two days off, so there was literally no time for me to finish (my course work). Even my clinical documentation, I have clinical documentation that we do in the hospital and then we must continue at home and then we have to submit.” |
|  |  | P6 | “Having more time for assignments is good, even if exams are more compact.” |
|  |  | P7 | “Having the three-day weekend helped me organize my time better.” |
|  |  | P10 | “And for my studies, now I can finish more during the weekend or on the days I come back.” |
|  |  | P11 | “Having an extra weekend day allowed me to balance both workload and commitments, helping me manage my time better.” |
|  |  | P13 | “It will help; since the implementation of the four-day week, the transfer probably increased, also having more energy after the weekend.” |
|  |  | P18 | “Can help you manage your time better. Help you do other things outside the academic club so you’re having more time to do with it.” P18 |
|  |  | P20 | “It’s improved. I can actually balance things now. For example, spending time with family because in dentistry we usually like almost every week, we have two to three exams usually and we have methods, and everything is just accumulated. So, like, if you mess up one exam, you’re going to lose marks at the end. So that’s why in the past, like I wouldn’t even be able to exit my room. I wouldn’t even see my parents. But now I can know it’s better.” |
| Support Systems | Faculty and family support | P2 | “It’s like I’ve been receiving motivation from others and motivating myself. Because I’m going through this, I now have to focus on myself more and spend time organizing priorities. I motivate myself, and my parents also motivate me.”  “Yes, starting from attending the university officially, I received (support) from doctor xxxx (a faculty member’s name). Every day I would go to her office, and I see her, and she knows me. I’m the problematic student, but she’s still like, tells me to take care and be careful and be a great student.” P2 |
|  |  | P3 | “Yes, from Doctor xxxx (a faculty member’s name) and Doctor xxxx (another faculty member’s name) as well; basically, in one of the semesters, I had Netflix shoot, and I was going to be absent from university for two months in a row. I talked with Doctor xxxx (a faculty member’s name) as she’s my advisor and she helped me out, also Doctor xxxx (another faculty member’s name) helped me and they made it possible for me to do my shooting, to do my work and to attend my makeup (exams) better.” |
|  |  | P5 | “Faculty members helped us with the lab timing and exam schedule, like for example, sometimes the client finish late in the clinic, so, we just start the lab like 30 minutes, 40 minutes late. And like sometimes when I have two quizzes in the same day or two midterms in and the same day, some faculty members, accept that I can do it in the other section. So that’s really supporting for the students because they think that there’s a chance and that is really helping us.” |
|  |  | P6 | “Yes, they (faculty) became more understanding for the situation. It was effective and we went to discuss the timing of quizzes or anything if they wanted to have extra classes on Sunday.” |
|  |  | P7 | “Yes, they made optional assignments. Also, during the exams of the last semester they opened one of the labs during Sunday.” |
|  |  | P12 | “They opened labs on Sundays, for example, during finals. Like if you want to go and revise. So, they give us one extra day from their time.” |
|  |  | P13 | “Yes, everyone from the faculty was supportive, and friends were also supportive.” |
|  |  | P14 | “Yes, faculty were motivating us, and family and friends also supported me.” |
| Classroom Dynamics | Same attendance levels | P1, P3, P4, P5, P6, P8, P9, P11, P12, P13, P16, P18, P21, P22, P23, & P24 | “Same (for five-day and four-day work weeks).” |
|  |  | P10 | “My attendance level is the same, there is no change.” |
|  |  | P17 | “My attendance level to lectures is less because it’s online, but for the labs, it’s same, no change.” |
|  | Improved attendance | P7 | “Slightly better attendance level compared to 5 weekdays.” |
|  |  | P19 | “My attendance got better; I’m attending all my classes now.” |
|  |  | P2, P14, P15, & P20 | “Better attendance level (for four-day week).” |
| Common stressors of a four-day academic week | Condensed schedules and long days | P1 | “The negatives of having four days are that the materials are compressed into these four days, leaving minimal time.” |
|  |  | P2 | “Ohh, I thought that it’s become overwhelming for a lot of students. Uh, one day has been removed from our schedule, which is Sunday. So, now we have to attend on Monday, Tuesday, Wednesday and Thursday, which makes the schedule more compact or more lectures back-to-back for. I feel that it’s more for medical students. Ohh, especially that they have lectures from 5 to 6 (PM), so even when they go back home or during their bus trip, they have to worry about opening the lecture online. So, it took time for me and probably for other students too.” |
|  |  | P3 | “The drawback is for example, nowadays the classes are many; let’s say for example today is Monday, so I started at 8:00 (AM) and I finished at 4.00 (PM), but before I used to start at 10 and then I would finish that too (at 4.00 PM). We have more time to divide the classes, but for me personally, and I think most people will agree with me, it’s fine to have everything compacted on the weekdays as long as on the weekend we have an extra day off.” |
|  |  | P4 | “The days became longer and compact. It became more stressful because the days became longer.” |
|  |  | P5 | “But on the other side, the drawbacks for having 4 days is (are) that we usually have a lot of materials that’s compressed on us in these four days that we have minimal time.” |
|  |  | P6 | “………… the side effects that we have - more condensed schedule during the day.” |
|  |  | P7 | “………………………. but there are more lectures, so your focus is a bit less.” |
|  |  | P9 | “The weekdays became more compressed, and the time management got harder.”  “The days became longer and compact, which is stressful.” |
|  |  | P12 | “I think the only drawback is longer working time. Because we never used to stay in the university from 10 to 4 or 5 o’clock. But for the benefits, is that we have more time to revise as I said.” |
|  |  | P15 | “The days became longer and compact. It became more stressful because the days became longer.” |
|  |  | P16 | “……………….. but the drawback is that the weekdays are compacted.” |
|  |  | P17 | “The schedule is compact, which is hard on students.” |
|  |  | P19 | “The only drawback is just the extra (work) hours (during the four-day week).” |
|  |  | P21 | “The benefit is having more time, but the drawback is that the weekdays are more condensed.”  “The lectures and labs per day increased, it became too much but it is manageable.”  “The only drawback is how everything is squeezed into this form.” |
|  |  | P22 | “The benefit is having more time, but a drawback is the more condensed days. The classes became more condensed, and it became harder to manage all the tasks within four days.” |
|  |  | P24 | “We had to try to adjust all the labs and lecture sessions within these four days, so it was very condensed but at the same time we got like three days off, which was a bit relaxing.” |
|  | Challenges | P1 | “…. but the drawbacks that we may forget things we may like that we will not study in these three days and sometimes it happened.” |
|  |  | P2 | “I’m arriving late to the lectures by around 15 minutes because of the back-to-back classes.”  “It’s very hard, there is extra load on me, I’m studying in the medical field that needs too much work, too much body stress, too much brainstorming, after all that, I try not to show my weakness to others, so I try to handle it, and when I go back home, I do my other stuff and I try my best to engage with my family also.” |
|  |  | P9 | “My grades got decreased due to stress.” |
|  |  | P10 | “……. but for the negative impact, as I mentioned, the tutors are unable to finish materials. So, there are a lot (of) self-study material.” |
|  |  | P14 | “So, very, very bad, very bad (in balancing academic workloads and responsibilities during four-day week).”  “It also entered our personal life, our commitments, responsibilities, so we don’t have life outside.” |
|  |  | P15 | “Worse balance between my responsibilities, family gatherings, personal time, and academic workload.” |
|  |  | P17 | “As an extra day in the weekend is a benefit, but as a drawback, it’s very hard for us.”  “Worse balance between my responsibilities, family gathering, personal time and my academic workload.” |
|  |  | P19 | “Very bad, it became worse. I felt like there was no time for my family and friends, because there was no time for me to study during weekdays, so I spend my weekends studying.”  “So, it was a very huge downgrade, even though I went back then when I used to have two days as a weekend, I actually get to spend more time with the family. But now since I need to finish this and finish that course of the week load, it has been pushed to the weekend. So, I do not have as much time.”  “The drawback is that everything is squeezed, leading to more self-study.”  “Benefits I get more time to study theoretical wise, but the drawback is clinical wise. I don’t always get the extra hours that I need, but if I’m going with the schedule of having my pre planned clinics, it’s actually perfect.” P19 |
|  |  | P20 | “….. but, for the drawbacks, the long hours can affect people’s mental health.” |
|  |  | P1, P4, P10, P11, P15, P16, P18, P19, P20, P21, P22, P23, & P24 | No (additional) support received from faculty. |

^a^P: participant.
